# Arabidopsis GPAT9 contributes to synthesis of intracellular glycerolipids but not surface lipids

**DOI:** 10.1093/jxb/erw242

**Published:** 2016-06-20

**Authors:** Stacy D. Singer, Guanqun Chen, Elzbieta Mietkiewska, Pernell Tomasi, Kethmi Jayawardhane, John M. Dyer, Randall J. Weselake

**Affiliations:** ^1^Agricultural Lipid Biotechnology Program, Department of Agricultural, Food and Nutritional Science, University of Alberta, Edmonton, Alberta T6G 2P5, Canada; ^2^USDA-ARS, US Arid-Land Agricultural Research Center, 21881 North Cardon Lane, Maricopa, AZ 85138, USA

**Keywords:** Acyl-CoA specificity, acyl lipid biosynthesis, cutin, GPAT, lipid droplet, pollen grain, regio-specificity, surface wax.

## Abstract

*GLYCEROL-3-PHOSPHATE ACYLTRANSFERASE* (*GPAT*) genes encode enzymes involved in glycerolipid biosynthesis in plants. Ten *GPAT* homologues have been identified in Arabidopsis. GPATs 4–8 have been shown to be involved in the production of extracellular lipid barrier polyesters. Recently, GPAT9 was reported to be essential for triacylglycerol (TAG) biosynthesis in developing Arabidopsis seeds. The enzymatic properties and possible functions of GPAT9 in surface lipid, polar lipid and TAG biosynthesis in non-seed organs, however, have not been investigated. Here we show that Arabidopsis GPAT9 exhibits *sn*-1 acyltransferase activity with high specificity for acyl-coenzyme A, thus providing further evidence that this GPAT is involved in storage lipid biosynthesis. We also confirm a role for GPAT9 in seed oil biosynthesis and further demonstrate that GPAT9 contributes to the biosynthesis of both polar lipids and TAG in developing leaves, as well as lipid droplet production in developing pollen grains. Conversely, alteration of constitutive *GPAT9* expression had no obvious effects on surface lipid biosynthesis. Taken together, these studies expand our understanding of GPAT9 function to include modulation of several different intracellular glycerolipid pools in plant cells.

## Highlight

Arabidopsis glycerol-3-phosphate acyltransferase 9 (GPAT9) is an *sn*-1 specific acyl-CoA:GPAT that contributes to intracellular glycerolipid biosynthesis in seeds, developing leaves and pollen grains, but not to extracellular glycerolipid biosynthesis.

## Introduction

Glycerolipids, including storage triacylglycerol (TAG), various polar membrane lipids, and numerous signaling molecules associated with plant growth, development, and resistance to biotic and abiotic stress, are produced in plants through three main biosynthetic pathways that are compartmentalized within plastids, mitochondria, and the endoplasmic reticulum (ER) (Roughan and Slack, 1982; Browse *et al.*, 1986). Glycerolipids produced within chloroplasts are converted mainly to galactolipids, which serve primarily as functional and structural components of photosynthetic membranes (Dörmann and Benning, 2002). Conversely, glycerolipids synthesized within mitochondria may contribute to the production of membrane lipids and the relative fatty acid (FA) composition of TAG in a number of plant organs (Zheng *et al.*, 2003). Glycerolipids produced on ER membranes generally constitute cellular membrane phospholipids, and in developing seeds are synthesized primarily as precursors in support of TAG production (Ohlrogge and Browse, 1995). Each of these three pathways consists of a distinct set of enzymes that essentially mediate the stereospecific esterification of FAs to a glycerol backbone, with an extensive coordinated exchange of glycerolipid molecules between the three compartments (Browse *et al.*, 1986; Kunst *et al.*, 1988).

Glycerol-3-phosphate acyltransferase (GPAT) provides a crucial function in these glycerolipid biosynthetic pathways, catalyzing the transfer of a fatty acyl moiety from acyl-CoA, dicarboxylic acid (DCA)-CoA or acyl-acyl carrier protein to *sn*-glycerol-3-phosphate (G3P) to form lysophosphatic acid (LPA). The resulting LPA is an important intermediate for the formation of numerous glycerolipids, including extracellular lipid polyesters, membrane lipids and storage TAG. The metabolic fate of LPA is determined in part by the subcellular localization of the GPAT enzyme, with a soluble form being present in the plastid stroma, and membrane-bound forms localized to the mitochondria and ER (Chen *et al.*, 2011*a*).

In Arabidopsis, ten *GPAT* genes, *ATS1* and *GPAT1-9*, have been identified to date (Nishida *et al.*, 1993; Zheng *et al.*, 2003; Beisson *et al.*, 2007; Gidda *et al.*, 2009). While *ATS1* appears to function in the production of major phospholipids that make up plastidial membranes (Kunst *et al.*, 1988), GPAT1 has been found to play an important role in the development of pollen grains (Zheng *et al.*, 2003). Interestingly, a recent study has shown that GPAT1 possesses *sn*-2 acyltransferase activity and can utilize DCA-CoA substrates, which indicates that it is likely involved in the biosynthesis of extracellular lipids (Yang *et al.*, 2012). Similarly, GPAT4–8 have also been found to exhibit *sn*-2 regio-specificity with a preference for DCA- or omega-OH-CoA as substrate and have been shown to play important roles in the production of the extracellular lipid barrier polyesters, cutin and/or suberin (Suh *et al.*, 2005; Beisson *et al.*, 2007; Li *et al.*, 2007*a*, *b*; Yang *et al.*, 2012).

In addition to the essential roles of GPATs in the production of extracellular lipid polyesters in plants, an ER-bound GPAT is believed to be crucial for the synthesis of storage TAG, catalyzing the first acylation reaction of the Kennedy pathway (reviewed by Bates *et al.*, 2013). Intriguingly, in castor (*Ricinus communis*), a homolog of Arabidopsis GPAT9 has been identified on purified ER membranes by proteomics (Brown *et al.*, 2012). Furthermore, GPAT9 exhibits the closest evolutionary relationship of all Arabidopsis GPATs to mammalian GPAT3 (Gidda *et al.*, 2009), which is known to play a crucial role in storage lipid biosynthesis (Cao *et al.*, 2006; Shan *et al.*, 2010). Indeed, both human GPAT3 and Arabidopsis GPAT9 contain an additional conserved motif above the four motifs that are typically characteristic of other GPAT enzymes (Lewin *et al.*, 1999). Unlike Arabidopsis *GPAT1–8*, homologs of *GPAT9* appear to be present in various algal species that also produce an abundance of TAG (Khozin-Goldberg and Cohen, 2011; Iskandarov *et al.*, 2015), and a recent study demonstrated that the *GPAT9* homolog from the oleaginous green microalga *Lobosphaera incisa* increased TAG content by up to 50% when heterologously expressed in *Chlamydomonas reinhardtii* (Iskandarov *et al.*, 2015). Additionally, it has recently been reported that GPAT9 plays a role in TAG biosynthesis in Arabidopsis, wherein down-regulation of *GPAT9* led to decreased seed oil content, and a *gpat9* mutant exhibited both male and female gametophytic lethality phenotypes (Shockey *et al.*, 2016).

However, while *GPAT9* appears to contribute to seed oil accumulation in Arabidopsis, there remains a lack of direct evidence that GPAT9, unlike other GPATs, is an *sn*-1 acyltransferase with a preference for acyl-CoAs as its substrate. Moreover, there is a paucity of information regarding its function in the biosynthesis of lipids in non-seed organs. In this study, we carried out *in vitro* enzyme assays using a yeast-based expression system and provide detailed functional investigations of Arabidopsis lines in which *GPAT9* was either overexpressed or down-regulated. Our findings indicate that GPAT9 exhibits *sn*-1 acyltransferase activity with high specificity for acyl-CoA, thus confirming its role in seed TAG biosynthesis, and provide comprehensive evidence in support of its role in the production of both polar and non-polar lipids in leaves, as well as lipid droplets in pollen. Furthermore, since GPAT1–8 have been reported to play important roles in the production of extracellular lipid barrier polyesters, we also investigated the possible contribution of GPAT9 to this process. The results of our studies not only yield a deeper understanding of the function of GPAT9, but also bring us closer to a full elucidation of intracellular and extracellular lipid biosynthesis in plants, which will be important for the future improvement of lipid content in oilseed crops.

## Materials and methods

### Heterologous expression of GPAT9 in yeast and *in vitro* enzyme assays

The full-length Arabidopsis *GPAT9* cDNA was inserted downstream of the *GAL1* promoter in the pYES2.1/V5-His-TOPO®TA yeast expression vector (Invitrogen) according to the manufacturer’s instructions. *Saccharomyces cerevisiae* strain *gat1Δ* (Matα, his3C1, leu2C0, lys2C0, ura3C0, YKR067w::kanMX4; Zheng and Zou, 2001) was transformed with both the experimental construct as well as the empty vector control, respectively. Microsomal fractions containing recombinant Arabidopsis GPAT9 were used in the enzyme assays.

While unsubstituted acyl-CoA substrates (16:0-CoA, 18:0-CoA, 18:1Δ^9*cis*^-(hereafter 18:1)-CoA, 18:2 Δ^9*cis,*12*cis*^-(hereafter 18:2)-CoA, and 18:3Δ^9*cis,*12*cis,*15*cis*^ (hereafter 18:3)-CoA were purchased directly (Avanti Polar Lipids, Inc.), 16:0-DCA-CoA was synthesized as described by Kawaguchi *et al.* (1981) and Yang *et al.* (2010). The *in vitro* GPAT enzyme assay was performed essentially as described previously (Yang *et al.*, 2010; Chen *et al.*, 2014).

### Plant growth conditions

Arabidopsis seeds were cold-treated at 4 ºC in the dark for 3–5 d prior to their placement in a growth chamber at 22 ºC. In most cases, plants were grown with a photoperiod of 18h day/6h night and 250 µmol m^−2^ s^−1^ light intensity for the remainder of their development. Plants utilized for wax analyses were grown in a growth chamber with a 12-h-light/ 12-h-dark cycle and a light intensity of approximately 700 µmol m^−2^ s^−1^. In all instances, plants were regularly watered and fertilized.

### RNA extraction, first-strand cDNA synthesis, and quantitative real-time RT-PCR

Various tissues were harvested from Arabidopsis plants (all within a Col-0 background) at a number of different developmental stages, flash frozen in liquid nitrogen, and stored at −80 ºC until further use. Total RNA was extracted using the Sigma Spectrum Plant Total RNA Kit (Sigma-Aldrich Canada Co., Oakville, ON) and contaminating DNA was removed using the TURBO DNA-free system (Ambion, Life Technologies Inc., Burlington, ON). First-strand cDNA synthesis was carried out using the Superscript III first-strand cDNA synthesis kit according to the manufacturer’s instructions (Invitrogen, Life Technologies Inc.).

Quantitative real-time RT-PCR assays were performed with SYBR green PCR master mix on an ABI 7900HT Fast Real-Time PCR System (Applied Biosystems). The primers are AtGPAT9_QF2 (5ʹ–CGG TGA AAC AGG AAT TGA ATT TG–3ʹ) and AtGPAT9_QR2 (5ʹ–AGA CCC GCC CGA AGA GAT A–3ʹ). Primers AtPP2AAF1 (5ʹ–TCA ATC CGT GAA GCT GCT GCA AAC–3ʹ) and AtPP2AAR1 (5ʹ–ACT GCA CGA AGA ATC GTC ATC CGA–3ʹ) were utilized to amplify a 146-nt fragment of the constitutively expressed *PROTEIN PHOSPHATASE 2A SUBUNIT 3* (*PP2AA3*) transcript (Czechowski *et al.*, 2005), which was utilized as an internal control. Levels of gene expression were obtained using the standard curve method and SDS v2.4 software (Applied Biosystems). The Arabidopsis Genome Initiative numbers for the gene sequences utilized are AT5G60620 (*GPAT9*) and AT1G13320 (*PP2AA3*).

### Generation of transgenic Arabidopsis

Five plant transformation constructs including one GPAT9-GUS (β-glucuronidase) translational fusion vector, two *GPAT9* overexpression vectors [constitutive (GPAT9-OE) and seed-specific (GPAT9-SS-OE), respectively], and two *GPAT9* RNAi vectors [constitutive (GPAT9-RNAi) and seed-specific (GPAT9-SS-RNAi), respectively], were generated.

The GPAT9-GUS vector was produced by first amplifying a 3345-bp fragment of the Arabidopsis *GPAT9* gene, along with primers AtGPAT9F4SalI (5ʹ–ATA **GTC GAC** GAG AAG ACG ACG AGA AGA GC–3ʹ) and AtGPAT9R4BamHI (5ʹ–AG**G GAT CC**C TCT GCG AAA CTC TGT TGC–3ʹ), which contain *Sal*I and *Bam*HI restriction sites near their 5ʹ ends, respectively (indicated in bold). This fragment was inserted upstream of the *GUSAint* sequence (encoding β-glucuronidase) with its start codon removed and *Nopaline synthase* transcriptional terminator (*nos-t*) to create an in-frame *GPAT9-GUS* translational fusion within the pGreen 0029 background (Hellens *et al.*, 2000).

To generate *GPAT9* overexpression constructs, the *GPAT9* coding sequence inserted between the constitutive tobacco *tCUP3* promoter (Wu *et al.*, 2003) and *Pisum sativum Ribulose-1,5-bisphosphate carboxylase* transcriptional terminator (*rbcS-t*) to generate the GPAT9-OE vector, or between the seed-specific *Brassica napus Napin* promoter (Josefsson *et al.*, 1987) and *rbcS-t* transcriptional terminator to produce the GPAT9-SS-OE vector.

To produce the *GPAT9* RNAi vectors, a 348-bp fragment near the 3ʹ end of the *GPAT9* coding region was amplified from the *GPAT9* cDNA clone with primers GPAT9RNAiF1SalI (5ʹ–**GTC GAC** GGG TGC TTT TGA ATT GGA CTG C–3ʹ) and GPAT9RNAiR1EcoRI (5ʹ–**GAA TTC** TCT TCC AAT CTA GCC AGG ATC G–3ʹ), as well as GPAT9RNAiF1XbaI (5ʹ–**TCT AGA** GGG TGC TTT TGA ATT GGA CTG C– 3ʹ) and GPAT9RNAiR1BamHI (5ʹ–**GGA TCC** TCT TCC AAT CTA GCC AGG ATC G–3ʹ) to generate sense and antisense copies. Although the *GPAT9* coding sequence displays virtually no significant similarities to any other Arabidopsis *GPAT* sequence (Gidda *et al.*, 2009), we further confirmed a lack of potential off-target effects by these fragments in Arabidopsis using the dsCheck software (http://dscheck.rnai.jp/). The *GPAT9* RNAi fragments were inserted in opposite orientations between the constitutive *tCUP3* promoter and intronic spacer from the pHannibal plasmid, and *rbcS-t* transcriptional terminator and intronic spacer, respectively. These same sense and antisense *GPAT9* fragments were also inserted between the seed-specific *Phaseolus vulgaris β-Phaseolin* promoter (Frisch *et al.*, 1995) and pHannibal intronic spacer, and the *β-Phaseolin* transcriptional terminator and intronic spacer, respectively, in a pPZP-RSC1 background (Hajdukiewicz *et al.*, 1994) to yield the GPAT9-SS-RNAi vector.

Vectors were introduced into *Agrobacterium tumefaciens* strain GV3101 via electroporation, and in the case of pGreen-derived vectors, the helper plasmid pSoup (Hellens *et al.*, 2000) was co-transformed simultaneously. The resulting recombinant bacteria were used for the transformation of Arabidopsis ecotype Col-0 using the floral dip method (Clough and Bent, 1998). The presence of target constructs in transgenic plants was confirmed by PCR and homozygous lines were identified using segregation analyses. For every experiment, transgenic experimental lines were grown in the same growth chamber at the same time as their corresponding negative control lines. In the case of all experiments involving T_1_ lines, plants transformed with empty vector were utilized as the wild-type control, while in experiments involving subsequent generations, wild-type controls comprised null-segregants.

### Morphological analyses and histochemical staining

T_3_ seed weights from homozygous transgenic lines and wild-type plants were calculated by first weighing small batches of seeds, followed by particle counting using a FuorChem SP Imager and AlphaEase software (Alpha Innotech Corp., San Leandro, CA). Three technical replicates were used in each case. T_3_ seed areas from the same lines were determined using the particle analysis function of ImageJ software (http://imagej.nih.gov/ij).

Various tissues from at least 15 independent T_1_ transgenic lines bearing the GPAT9-GUS vector were stained for GUS activity essentially as described by Jefferson *et al.* (1987). Images were obtained using an Olympus SZ61 microscope with attached digital camera (Olympus Canada Inc., Richmond Hill, ON).

Toluidine blue staining for estimation of cuticle permeability was carried out by dipping rosette leaves [28 d after planting (DAP)] and open flowers from 10 GPAT9-RNAi T_2_ lines and wild-type plants, respectively, into 0.05% (w/v) toluidine blue for 2min at room temperature, followed by a rinse with distilled water. Pollen staining was conducted with anthers from 10 T_2_ GPAT9-RNAi lines, multiple plants from two T_3_ GPAT9-RNAi lines, and wild-type plants according to the method described by Peterson *et al.* (2010).

### Lipid extraction and GC-MS analyses

Arabidopsis seed lipid extractions were carried out using mature seeds from GPAT9-OE, GPAT9-SS-OE, GPAT9-RNAi, GPAT9-SS-RNAi and wild-type plants as described in Pan *et al.* (2013). Total leaf lipids were extracted from developing GPAT9-OE, GPAT9-RNAi, and wild-type leaves (35 DAP). In each case, two leaves (leaves 5 and 6) from three individual T_3_ plants were utilized for lipid extractions using the protocol described by the Kansas Lipidopmics Research Center (https://www.k-state.edu/lipid/lipidomics/leaf-extraction.html). Internal standards of 0.0025mg 17:0 TAG and 0.05mg 19:0 phosphatidylcholine (PC) (Avanti Polar Lipids Inc., Alabaster, AL) were included in each reaction. Leaf lipids were separated into lipid classes by one-dimensional TLC on silica gel plates (SIL G25, 0.25mm, Macherey-Nagel, Düren, Germany) using hexane/diethyl ether/glacial acetic acid (70:30:1, v/v) as the solvent as described by Mietkiewska *et al.* (2011). Lipid classes were visualized under UV irradiation following treatment with 0.05% (w/v) primuline and spots corresponding to TAG and polar lipids (PLs) were scraped off and transmethylated with 3N methanolic HCl at 80 ºC for 1h. The resulting FA methyl esters were extracted twice with hexanes, evaporated under a stream of nitrogen gas and resuspended in 500 µl iso-octane with methyl heneicosanoin (21:0 methyl ester, 0.1mg/ml; Nu-Chek Prep Inc.) as a second internal standard.

All extracted FA methyl esters were analyzed using an Agilent 6890 Network GC system equipped with a DB-23 capillary column (30 m × 0.25mm × 0.25 µm) and a 5975 inert XL Mass Selective Detector (Agilent Technologies Canada Inc., Mississauga, ON). The following temperature program was utilized: 100 ºC, hold for 4min, 10 ºC min^−1^ to 180 ºC, hold for 5min, and 10 ºC min^−1^ to 230 ºC, hold for 5min.

### Transmission electron microscopy

To visualize morphological changes in the pollen of GPAT9-OE and GPAT9-RNAi lines compared to wild-type plants, flower buds were fixed with 2.5% gluteraldehyde and 2% paraformaldehyde in 0.1M phosphate buffer (pH 7.2–7.4) at room temperature overnight and then stored at 4 ºC. Fixed samples were then post-fixed in 1% (w/v) osmium tetroxide in 0.1M phosphate buffer (pH 7.2–7.4) for 3h at room temperature. The samples were then dehydrated in a graded ethanol series, infiltrated with propylene oxide, and embedded using the Spurr’s Low Viscosity embedding kit (Electron Microscopy Sciences, Hatfield, PA). Ultrathin (80nm) sections were generated using an Ultracut E ultramicrotome (Reichert Inc., Depew, NY) and were stained with 4% uranyl acetate and lead citrate solution (Reynolds, 1963) for TEM microscopy using a Morgagni 268 TEM microscope (FEI Company, Hillsboro, OR) at an accelerating voltage of 80kV. Images were photographed using an attached Orius CCD camera (Gatan Inc., Pleasanton, CA).

### Analysis of epicuticular waxes

Surface waxes were extracted and analyzed as described previously (Jenks *et al.*, 1995) with slight modifications. In brief, each sample was submerged in 10ml hexanes and gently agitated for 45s. The samples were then removed from the solvent with forceps and placed on a transparency sheet for scanning. Three internal standards (10 µg of nonadecanoic acid, 10 µg of tetracosane and 20 µg of pentacosanol) were added to each sample vial. Wax extracts were heated at 60 °C then the solvent was reduced under N_2_ until the volume could be transferred into a 2ml glass vial. The scintillation vials were rinsed once with hexanes, the volume transferred again, and then evaporated to dryness.

For each wax sample, 100 µl of N, O-bis (trimethylsilyl) trifluoroacetamide (BSTFA) and 100 µl hexanes were added for a total volume of 200 µl. The samples were analyzed on an Agilent 7890A gas chromatograph equipped with a 5975C mass spectrometer and an onboard heater and shaker. Four leaves were harvested from five replicate plants 35 DAP, while stem samples were collected from the same plants 42 DAP. Surface areas were determined from sample scans using ImageJ. Each leaf value was multiplied by two to account for both surfaces and stem scans were multiplied by π. Quantified wax values are expressed as µg dm^−2^. Least squares means (*P*<0.05) were calculated for statistical analysis (*P*<0.05).

### Isolation and analysis of cutin

Cutin monomers from Arabidopsis leaves harvested 28 DAP were isolated and analyzed as described by Bonaventure *et al.* (2004) and Chen *et al.* (2011*b*). GC-MS analysis was performed using an Agilent 6890N gas chromatograph with an Agilent 5975 Inert Mass Selective Detector as described previously (Chen *et al.*, 2011*b*). Chromatographic separation was achieved using an HP-5MS capillary column (30 m, 0.25mm, 0.25mm; Agilent Technologies) with a temperature program of 140 ºC to 300 ºC at a rate of 3ºC min^−1^. The inlet was operated in split mode (10:1 split ratio, 1ml injection) at 310 ºC. For the mass spectra conditions, the solvent delay was 4min, ionization energy was 70eV, and data were acquired in scan mode with a range of 35 to 500 atomic mass units.

## Results

### Arabidopsis GPAT9 is an sn-1 acyltransferase with high specificity for acyl-CoA

**Fig. 1.**
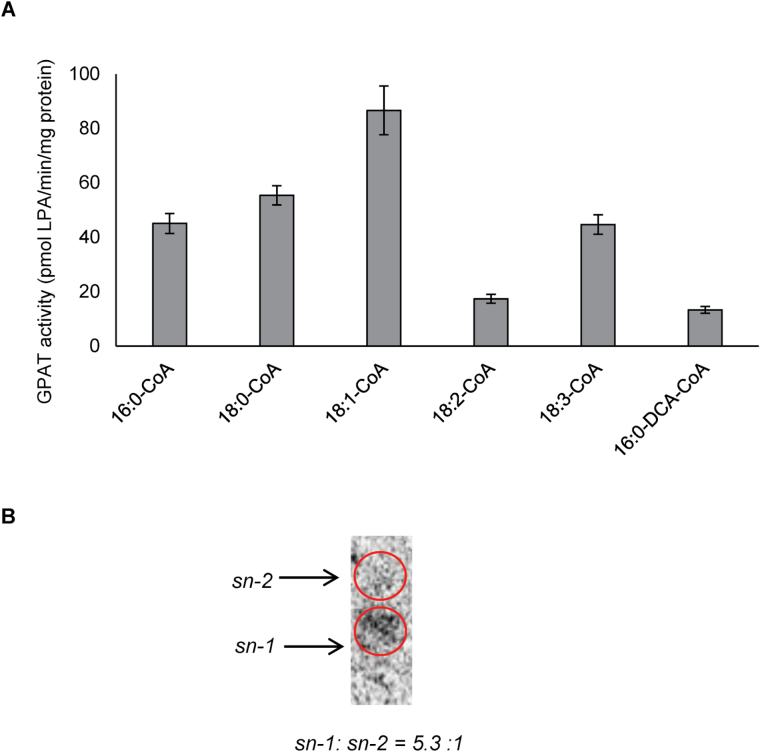
Substrate specificity and regio-specificity of Arabidopsis GPAT9. (A) *In vitro* enzyme assays of GPAT9 indicate that it prefers acyl-CoA over dicarboxylic acid (DCA)-CoA as a substrate. Bars represent mean enzyme activities when the enzyme was assayed at 22 ºC for 20min. Standard errors of three independent enzyme preparations are also shown. Microsomes were prepared from yeast cells. *Saccharomyces cerevisiae* strain *gat1Δ* (Matα, his3C1, leu2C0, lys2C0, ura3C0, YKR067w::kanMX4; Zheng and Zou, 2001) was transformed with Arabidopsis *GPAT9* and an empty vector control, respectively. (B) *In vitro* regio-specificity assays of GPAT9 reveal the enzyme is an *sn-1* acyltransferase. Regio-specificity was determined by de-phosphorylating the lysophosphatidic acid (LPA) product, generated in the enzyme assays, using a commercial *E. coli* alkaline phosphatase and subsequently separating the resulting *sn*-1 and *sn*-2 monoacylglycerol on a borate-TLC plate.

The *in vitro* enzyme assays of recombinant GPAT9 were initially carried out at 22 ºC using 16:0-CoA and G3P as substrate for 0, 5, 10, 20 and 60min yielding 0, 213.4±11.0, 389.2±20.5, 900.5±42.7 and 2622.7±65.0 pmol LPA/mg protein (corrected in each case for residual background activity in empty vector controls), respectively, indicating that GPAT9 is indeed a functional GPAT enzyme. Subsequent assays using various other substrates, including different molecular species of acyl-CoA and 16:0-DCA-CoA, further demonstrated that GPAT9 prefers acyl-CoAs over 16:0-DCA-CoA as its substrate (Fig. 1A), with 18:1-CoA resulting in the highest activity of all acyl-CoA substrates tested. GPAT9 was not found to possess any phosphatase activity, since monoacylglycerol was not produced in the reaction mixtures of these assays. Analysis of the regio-specificity of GPAT9 indicated that the majority of the acylation reactions catalyzed by this enzyme took place at the *sn*-1 position rather than the *sn*-2 position (5.3:1 ratio; Fig. 1B). Taken together, the preference of GPAT9 for acyl-CoA as its substrate, and its *sn*-1 regio-specificity, provide support that it plays an important role in the Kennedy pathway of glycerolipid biosynthesis.

### GPAT9 is expressed throughout Arabidopsis development

**Fig. 2.**
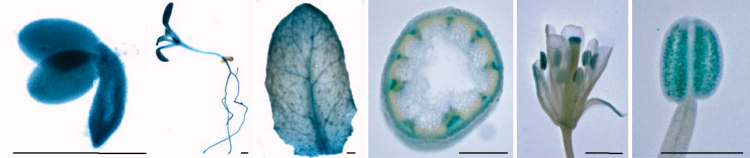
GUS staining of transgenic GPAT9-GUS translational fusion lines. Pictures are representative of at least 15 independent T_1_ lines. From left to right, tissues include a developing embryo, young seedling, rosette leaf, stem cross-section, flower, and anther. Scale bars, 10 μm.

Both quantitative real-time RT-PCR analyses and GUS staining of GPAT9-GUS translational fusion lines indicated that the Arabidopsis *GPAT9* gene is expressed in a relatively constitutive manner (Fig. 2, Supplementary Fig. S1at *JXB* online). Interestingly, the expression pattern of *GPAT9* appears to be much broader than that of *LYSOPHOSPHATIDIC ACID ACYLTRANSFERASES*, *DIACYLGLYCEROL ACYLTRANSFERASES* (*DGAT*s), *PHOSPHOLIPID:DIACYLGLYCEROL ACYLTRANSFE RASE,* and *PHOSPHATIDIC ACID PHOSPHATASES* when compared to previous microarray data (Supplementary Table S1). Indeed, comparable levels of *GPAT9* expression were noted in the majority of tissues tested, including seedlings, rosette and cauline leaves (28 DAP), stems, roots, floral buds, open flowers, pollen, and siliques/seeds/embryos at various developmental stages. The highest levels of expression appeared to be evident in leaves and developing siliques [at ~9 d after flowering (DAF)]. Within siliques, developing embryos displayed strong GUS staining in the mid-stages of embryo development, during the time of abundant glycerolipid biosynthesis in this tissue. Within stem cross-sections, GUS staining was apparent in both the phloem and xylem, but appeared to be weak or absent in epidermal cells. In floral tissues, the majority of *GPAT9* expression was restricted to anthers (and more specifically pollen), while little or no GUS staining was observed in sepals or petals. This expression profile of *GPAT9* in floral tissues is not entirely consistent with the relatively constitutive expression pattern observed in previous microarray assays (Schmid *et al.*, 2005; Winter *et al.*, 2007), which may reflect differences in the timing/developmental stage used in the GUS staining experiments. It is also possible that the promoter fragment utilized for our GUS assays lacks certain *cis*-acting elements present in the native gene context.

### Validation of constitutive GPAT9 overexpression and RNAi Arabidopsis lines

**Fig. 3.**
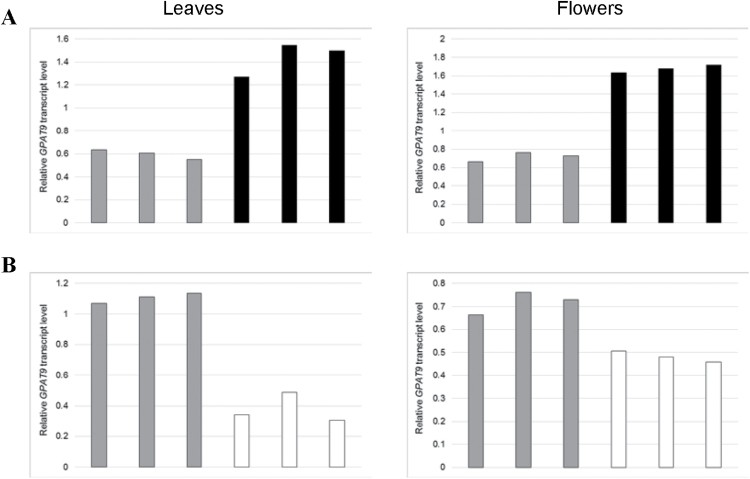
Confirmation of alterations in constitutive *GPAT9* expression in leaves and flowers of GPAT9-OE and GPAT9-RNAi lines compared to wild type. Quantitative real-time RT-PCR analysis of *GPAT9* expression in independent T_1_ GPAT9-OE (A), GPAT9-RNAi (B) and wild-type lines. Blocks denote mean *GPAT9* transcript levels from three technical replicates relative to the internal control, *PP2AA3*. Gray blocks represent wild-type plants, black blocks represent independent GPAT9-OE lines, and white blocks represent independent GPAT9-RNAi lines.

Transgenic lines constitutively overexpressing or down-regulating *GPAT9* in Arabidopsis were generated (Supplementary Fig. S2) to elucidate the function of this gene at various stages of plant development *in planta*. Initially, leaf and floral tissues were harvested from a selection of independent T_1_ lines, as well as wild-type plants, and levels of *GPAT9* transcripts were assayed using quantitative real-time RT-PCR. In the case of GPAT9-OE lines, *GPAT9* transcripts were increased by between 113% and 159% (leaf tissue), and 128% and 139% (floral tissue), compared to wild type, while in GPAT9-RNAi lines, *GPAT9* transcripts were reduced by between 56% and 72% (leaf tissue), and 30% and 36% (floral tissue), compared to wild type (Fig. 3). Furthermore, developing T_2_ siliques containing T_3_ seeds (14 DAF) were also harvested from two independent transgenic GPAT9-OE and GPAT9-RNAi lines (utilized throughout this study), respectively, and following the identification of homozygous lines in each case, appropriate alterations in *GPAT9* expression were also confirmed in this tissue type (Supplementary Fig. S3). In GPAT9-OE siliques, *GPAT9* transcripts were increased by 127% (GPAT9-OE-6) and 73% (GPAT9-OE-7) compared to wild-type levels, while in GPAT9-RNAi siliques, *GPAT9* transcripts were diminished by 34% (GPAT9-RNAi-10) and 52% (GPAT9-RNAi-21) compared to wild-type lines (Supplementary Fig. S3). No obvious morphological changes were observed visually in vegetative or floral tissues from any independent line of any generation. Furthermore, neither germination (which occurred within 24h in all transgenic lines and wild-type plants) nor growth rates were altered in transgenic lines (Supplementary Fig. S9).

### Overexpression and down-regulation of GPAT9 in Arabidopsis results in changes in seed size, as well as seed oil content and composition

To gain insight into the possible role of *GPAT9* in Arabidopsis seeds, we subjected homozygous T_3_ GPAT9-OE and GPAT9-RNAi seeds to an in-depth analysis of seed morphology. Interestingly, relative mean seed weights were increased by 13.4% (GPAT9-OE-6) and 9.4% (GPAT9-OE-7) in the GPAT9-OE lines, while GPAT9-RNAi lines exhibited an 18.7% (GPAT9-RNAi-10) and 11.0% (GPAT9-RNAi-21) reduction in seed weight compared to wild type (Supplementary Fig. S4A). In line with this, mean seed areas in GPAT9-OE lines were increased 8.4% (GPAT9-OE-6) and 2.2% (GPAT9-OE-7), while GPAT9-RNAi lines displayed mean seed areas that were decreased by 10.3% (GPAT9-RNAi-10) and 9.7% (GPAT9-RNAi-21) compared to wild-type seeds (Supplementary Fig. S4B). No significant differences were noted in seed yields between either T_3_ homozygous GPAT9-OE (255.3mg/plant ±28.7 SE, *n*=12) and wild-type (253.7mg/plant ±17.1 SE, *n*=14) lines, or GPAT9-RNAi (304.4mg/plant ±12.1 SE, *n*=12) and wild-type (330.8mg/plant ±18.0 SE, *n*=14) lines.

Similarly, while overexpression of *GPAT9* in GPAT9-OE lines resulted in increased seed oil content, down-regulation of this gene in GPAT9-RNAi lines yielded significant decreases in seed oil content (Supplementary Figs S5–S8). To ascertain that any changes in lipid production in transgenic seeds were not a result of phenotypic alterations in vegetative or floral tissues, we also produced lines that overexpressed or down-regulated *GPAT9* in a seed-specific manner (GPAT9-SS-OE and GPAT9-SS-RNAi; Supplementary Fig. S2) and confirmed these alterations in expression via qRT-PCR (Supplementary Fig. S3). As was the case for constitutive lines, both seed-specific overexpressing and RNAi lines exhibited small but significant increases and decreases in oil content, respectively, compared to wild-type seeds (Supplementary Figs S5–S8). Furthermore, alterations in seed oil composition were evident in both overexpression and RNAi lines, with overall increases in 18:1 and 22:0, and decreases in 20:1 in overexpression lines (Supplementary Figs S5, S6) and increases in C18:0, C18:1 and C18:3, and reductions in 16:0, 18:2, 20:1, 22:0 and 22:1 in RNAi lines (Supplementary Figs S7, S8). These results are consistent with a recent report of *GPAT9* down-regulation via amiRNA (Shockey *et al.*, 2016). Further details regarding these seed-based results can be found in Supplementary Data S1.

### Overexpression of GPAT9 in Arabidopsis increases triacylglycerol and polar lipid levels in leaves

**Fig. 4.**
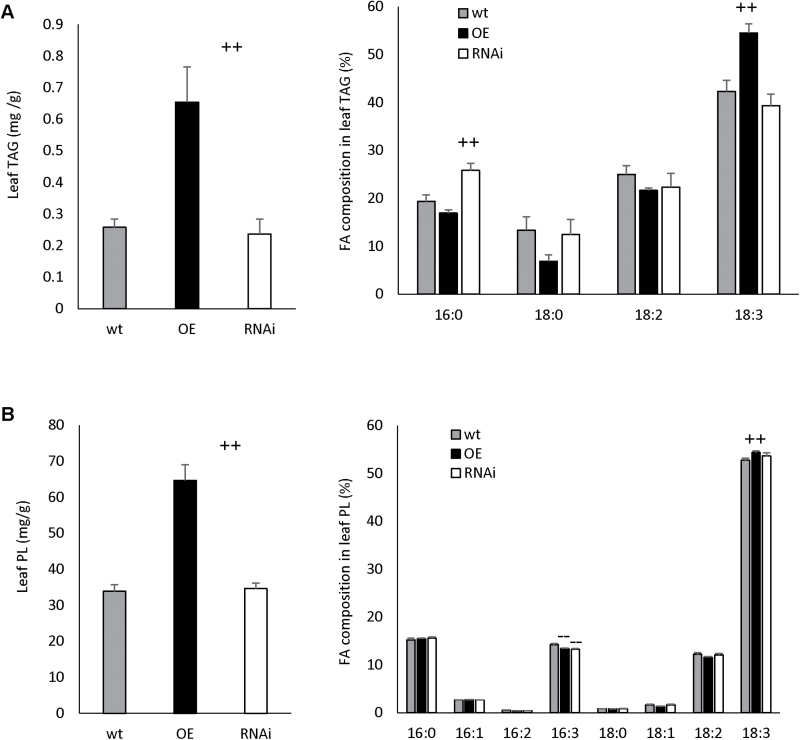
Lipid content and composition in constitutive GPAT9-OE, GPAT9-RNAi and wild-type 35 DAP rosette leaves. (A) Triacylglycerol (TAG) content and fatty acid composition. (B) Polar lipid (PL) content and fatty acid composition. Blocks represent the mean values of three biological replicates from two independent homozygous T_3_ lines in each case. Bars denote standard errors. Significant increases and decreases compared to wild type (as measured by Student’s *t*-test) are indicated by ++ and - -, respectively (*P*≤0.01). DW, dry weight; FA, fatty acid; OE, GPAT9-OE lines; RNAi, GPAT9-RNAi lines; wt, wild type.

Leaf lipids were analyzed to gain insight to the possible role of GPAT9 in lipid biosynthesis in non-seed organs. No significant differences were observed in the levels of either TAG or PL in GPAT9-RNAi lines compared to wild-type plants. On the contrary, GPAT9-OE leaves exhibited a 153.3% relative increase in TAG and a 91.0% relative increase in PL compared to wild-type plants (Fig. 4).

In addition to increases in the lipid content of GPAT9-OE leaves, alterations in the FA composition of both lipid classes were also observed in these lines. In the case of TAG, a significant 28.8% relative increase in 18:3 was observed in GPAT9-OE lines compared to wild type, with concomitant decreases in 16:0 (12.4%), 18:0 (48.6%) and 18:2 (13.3%; although these three latter changes were not significant). In leaf PL of GPAT9-OE lines, there was a significant decrease of 5.5% relative to wild type in 16:3Δ^7*cis,*10*cis,*13*cis*^ and a significant increase of 3.1% in 18:3. GPAT9-RNAi lines also displayed changes in FA composition within leaf tissues, with a significant increase in 16:0 (33.6%) in leaf TAG and a significant decrease in 16:3 (6.7%) in PL relative to wild-type plants (Fig. 4).

### Altering the constitutive expression of GPAT9 in Arabidopsis enhances production of lipid droplets in pollen grains

**Fig. 5.**
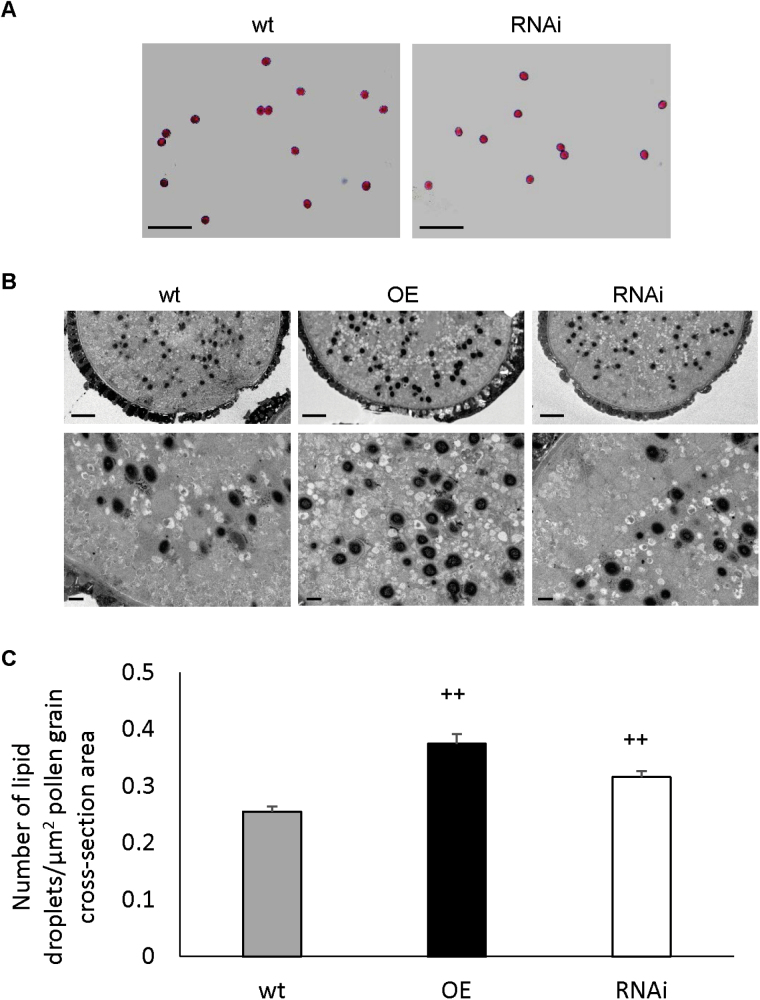
Effect of constitutive *GPAT9* overexpression and down-regulation on pollen development in Arabidopsis. (A) Alexander staining of pollen derived from homozygous wild-type and GPAT9-RNAi lines indicates that pollen viability was not affected by down-regulation of *GPAT9*. Scale bars, 100 μm. (B) Representative TEM micrographs of sectioned pollen grains derived from homozygous GPAT9-OE, GPAT9-RNAi and wild-type lines. As osmium tetroxide was utilized as a staining agent, lipid droplets are apparent as dark spots. Scale bars, 2 μm (top panel) and 0.5 μm (bottom panel). (C) Number of lipid droplets per square unit area of pollen grain cross-section. Blocks represent mean values from GPAT9-OE (*n*=29), GPAT9-RNAi (*n*=36) and wild-type (*n*=21) pollen grains obtained from at least three separate plants in each case. Bars indicate standard errors. Significant increases compared to wild-type (as measured by Student’s *t*-test) are indicated by ++ (*P*≤0.01). OE, GPAT9-OE lines, RNAi, GPAT9-RNAi lines; wt, wild-type.

Since knockout of *gpat9* has been shown to result in a male gametophytic lethality phenotype (Shockey *et al.*, 2016) and *GPAT9* has a high expression level in pollen (Fig. 2), we further hypothesized that GPAT9 may contribute to lipid biosynthesis in pollen grains. Since no differences were observed in the fertility of GPAT9-RNAi lines compared to wild type in this study, we further conducted Alexander staining (Alexander, 1969) of pollen grains from homozygous constitutive GPAT9-RNAi and wild-type plants to ensure pollen viability was not subtly affected by down-regulation of *GPAT9* expression. As expected, pollen from both GPAT9-RNAi and wild-type plants appeared morphologically normal and exhibited pink staining, confirming viability of the pollen grains in both cases (Fig. 5A).

To gain a deeper understanding of a possible role for GPAT9 in lipid biosynthesis within pollen grains, which are known to accumulate relatively high levels of TAG (Murphy, 2005), we also carried out TEM of pollen sections from homozygous constitutive GPAT9-OE, GPAT9-RNAi and wild-type lines. Examination of the resulting micrographs revealed pollen that was undistinguishable in morphology between both transgenic and wild-type lines, with no obvious alterations in the production of the extracellular exine lipid layer (Fig. 5B). Interestingly, the number of internal lipid droplets observed per unit area of pollen grain cross-section was significantly increased in both GPAT9-OE (46.9% relative increase) and GPAT9-RNAi (24.0% relative increase) lines compared to wild type (Fig. 5C); however, the increase detected in the overexpression lines was significantly greater than that observed in the RNAi lines. In GPAT9-OE pollen, lipid droplets also appeared slightly larger on average than in wild-type or RNAi plants (Fig. 5B), although we found that accurate measurements of droplet areas were not possible due to the fact that their borders were not sufficiently well-defined. Furthermore, while lipid droplets in wild-type lines were generally absent in a band near the outer edges of the grain, those from overexpression lines tended to accumulate indiscriminately throughout the pollen grain (Fig. 5B). Taken together, these results show that perturbation of *GPAT9* through either overexpression or knockdown disrupts normal lipid droplet accumulation in pollen grains.

### Cuticular and epicuticular lipids are not affected in constitutive GPAT9-OE or GPAT9-RNAi lines

**Fig. 6.**
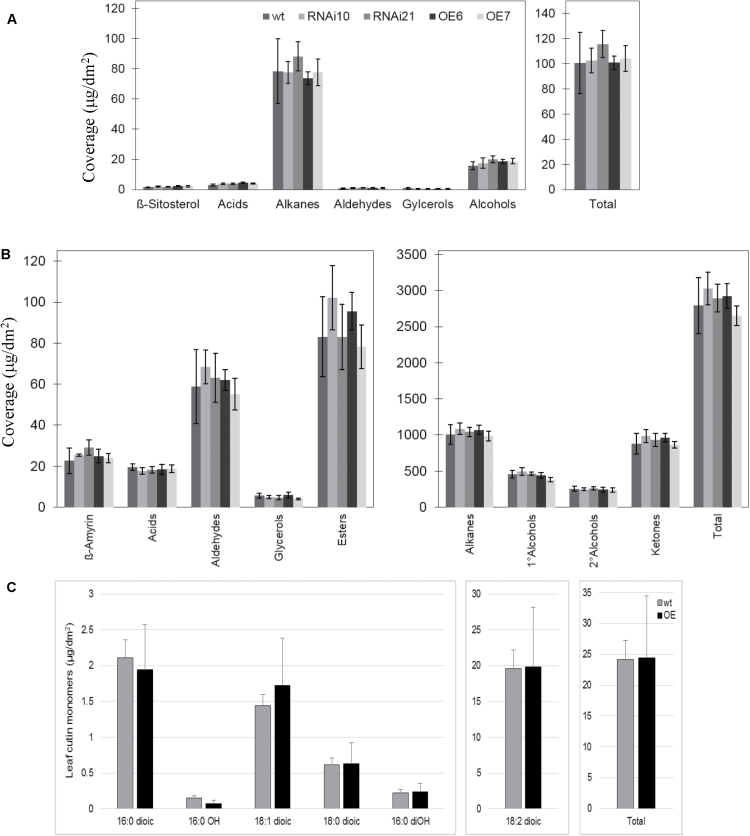
Epicuticular wax and cutin monomer content and composition of wild-type Arabidopsis and Arabidopsis with modulated constitutive *GPAT9* expression. (A) Epicuticular wax profile of leaves of wild-type (wt), GPAT9-RNAi (RNAi), and GPAT9-OE (OE) Arabidopsis. Data are presented as average ±standard deviation of five replicates. (B) Epicuticular wax profile of stem of wt, RNAi, and OE Arabidopsis. Data are presented as average±standard deviation of five replicates. C, Leaf cutin monomers from WT and OE lines. Two technical replicates were carried out in each case. Blocks represent mean values from three biological replicates and bars denote standard errors.

In order to determine whether there was any obvious reduction in cuticular lipids in constitutive GPAT9-RNAi lines, we carried out toluidine blue staining of both rosette leaves (28 DAP) and open flowers. This method is utilized extensively to examine cuticle defects in plant tissues (Tanaka *et al.*, 2004). No staining was observed in either wild-type or GPAT9-RNAi lines, indicating that permeability of the cuticular layer was not reduced to any appreciable extent in the RNAi lines in either leaves or floral tissues (data not shown). In agreement with these observations, extraction and biochemical analysis of epicuticular wax components from leaves and stems showed no obvious changes in content or composition between wild-type and GPAT9-RNAi lines (Fig. 6A, B, respectively, *P*<0.05). Furthermore, no obvious changes in either surface waxes or cutin monomers were observed in plant lines constitutively overexpressing *GPAT9* (Fig. 6C; *P*<0.05). Taken together, these data reveal that modulation of *GPAT9* expression has no obvious effects on the production of lipids that are destined for the plant surface.

## Discussion

The ER-localized Kennedy pathway for the synthesis of glycerolipids is believed to play a key role in the production of TAG and membrane lipids in most organisms. The first acylation step of this pathway is believed to be catalyzed by an ER-bound *sn*-1 regio-specific GPAT with a preference for acyl-CoA as its substrate in various organisms; however, there has been much debate regarding the particular enzyme that catalyzes this reaction in plants (Chen *et al.*, 2015). Very recently, knockdown of Arabidopsis *GPAT9* has been found to result in reduced seed oil content (Shockey *et al.*, 2016), which suggests that this gene is involved in seed TAG biosynthesis in plants. However, direct evidence for such a role in the form of enzymatic activity, as well as its function in non-seed tissues, is lacking. As such, we have carried out an in-depth characterization of this gene and the enzyme it encodes in an attempt to gain further insight into its function in plants.

Unlike all previously characterized Arabidopsis GPAT enzymes, which demonstrate *sn*-2 regio-specificity and a preference for omega-oxidized-acyl-CoAs as their substrates (Yang *et al.*, 2012), our *in vitro* enzyme assays revealed that GPAT9 exhibits *sn*-1 regio-specificity and a preference for acyl-CoAs rather than DCA-CoA as a substrate (Fig. 1B). These results provide direct evidence that GPAT9 catalyzes the first step in the Kennedy pathway and regulates the *in vivo* biosynthesis of intracellular lipids in Arabidopsis. Furthermore, phosphatase activity has been confirmed previously in Arabidopsis GPAT4, GPAT6 (Yang *et al.*, 2010) and GPAT8 (Yang *et al.*, 2012), resulting in the conversion of a proportion of the LPA product to monoacylglycerol, which has been suggested to be important for polyester assembly (Yang *et al.*, 2010). However, *in silico* analysis of the deduced GPAT9 amino acid sequence revealed that several amino acids known to be essential for this phosphatase activity in other plant GPATs, including DxD residues in motif I (DxD[T/V][L/V]) and K-D-D residues in motif III (K-[G/S][D/S]xxx[D/N] (Yang *et al.*, 2010), were altered in GPAT9. Further confirmation of the lack of phosphatase activity was obtained via *in vitro* GPAT9 enzyme assays, and is in line with expectations for a GPAT involved in the Kennedy pathway.

It has been suggested previously that the plant GPAT responsible for the first acylation step of the Kennedy pathway might not exhibit a strong substrate preference, and instead acyl-CoA substrate availability might determine the acyl composition of the *sn*-1 position of TAG (Snyder *et al.*, 2009). Conversely, in this study, our enzyme assay data indicate that of the acyl-CoA substrates tested, GPAT9 exhibited the highest activity with 18:1-CoA (Fig. 1A). This correlated well with our GPAT9-OE and GPAT9-SS-OE seed lipid data (Supplementary Figs S5, S6), which also showed consistent significant increases in the proportion of 18:1 compared to wild-type plants. Taken together, these findings imply that Arabidopsis GPAT9 enzymes may indeed display preferences for certain FA substrates. A comparative study of substrate specificity and selectivity of GPAT9s from diverse oilseed plants and oleaginous microalgae (such as the recently reported *L. incisa* GPAT9-like GPAT; Iskandarov *et al.*, 2015) could further expand our understanding of the role of these enzymes in TAG biosynthesis and their potential application for the future improvement of oilseed crop quality.

As both our quantitative real-time RT-PCR and GUS fusion results indicated that *GPAT9* is expressed at relatively high levels in developing leaves (Fig. 2, Supplementary Fig. S1), we sought to understand its possible function in this tissue type by analyzing the production of both extracellular and intracellular lipids in GPAT9-OE and GPAT9-RNAi lines. In line with our *in vitro* enzyme assay results, quantification of cutin monomer and epicuticular wax content and composition in transgenic lines indicated that alteration of *GPAT9* transcript levels did not have any effect on the production of surface lipids (Fig. 6). This, along with the fact that we did not detect significant amounts of *GPAT9* expression in epidermal cells (Fig. 2), implies that GPAT9 does indeed have a very different function in lipid biosynthesis from the previously characterized GPAT1 and GPAT4–8 enzymes.

In plant leaves, intracellular lipids are synthesized via two complementary pathways associated with the chloroplast (prokaryotic pathway) and ER (eukaryotic pathway), and mainly comprise thylakoid membrane galactolipids and other polar membrane lipids. A very small proportion of TAG is also generated in leaf tissues and is thought to participate in carbon storage and/or membrane lipid remodeling, but as of yet, its exact role remains elusive (Murphy and Parker, 1984; Murphy, 2001; Kaup *et al.*, 2002; Lin and Oliver, 2008; Slocombe *et al.*, 2009).

Previously, mutation or overexpression of the final ER-bound acyltransferase in the Kennedy pathway (DGAT) has been shown to affect the levels of TAG in leaves (Bouvier-Navé *et al.*, 2000; Slocombe *et al.*, 2009), which hinted at the possibility that this may also be the case for GPAT9. Indeed, in 35 DAP leaves from GPAT9-OE lines, we found a significant and substantial increase in both PL and TAG levels compared to wild-type lines (Fig. 4), suggesting that GPAT9 contributes to TAG and membrane lipid biosynthesis in Arabidopsis leaves. Since both PL and TAG would be expected to be derived, at least in part, from pathways that initiated with the *sn*-1 acylation of G3P by an ER-bound GPAT, it is not surprising that both lipid fractions were increased in transgenic overexpression lines. Since polar lipids, including galactolipids and phospholipids, have important roles in leaf development and function, GPAT9 activity may also affect the cellular and physiological performance of leaves. Therefore, further analysis of various subclasses of polar lipids, including galactolipids, in GPAT9-OE leaves would be an interesting next step in future investigations of GPAT9 function in leaf development.

We also observed a significant increase in 18:3 and concomitant decrease in 16:3 in the PL fraction from GPAT9-OE leaves compared to wild type, which strongly implied a shift towards the eukaryotic pathway (18:3) from the prokaryotic pathway (16:3), providing further confirmation that GPAT9 plays a role in the ER-localized eukaryotic glycerolipid biosynthesis pathway (Fig. 4B). Interestingly, a similar phenomenon was observed in a previous study when the plastidial Arabidopsis *GPAT* (*ATS1*), which is involved in the prokaryotic pathway of leaf lipid biosynthesis, was knocked out in Arabidopsis: the mutant plant was essentially converted from a 16:3 to a 18:3 plant (Kunst *et al.*, 1988).

While no significant differences were noted in the levels of TAG or PL in GPAT9-RNAi leaves compared to wild type, FA compositional changes were evident in both lipid types (Fig. 4). Since *GPAT9* is expressed at moderate levels in a relatively constitutive manner (Fig. 2), but knockout is known to be lethal (Shockey *et al.*, 2016), it appears that down-regulation of *GPAT9* transcripts by 34% and 52% yields enough GPAT9 activity to maintain normal plant morphology and lipid production. It is also possible that other enzymes, or perhaps an alternative lipid biosynthetic pathway, contribute in the compensation for down-regulation of *GPAT9* in leaf tissues.

Interestingly, the changes in fatty acid composition in PL from both GPAT9-OE and GPAT9-RNAi leaves were very small compared to those observed in TAG. Since fatty acid composition in PL can significantly affect the function of cell membranes, and there is substantial interchange of polar lipids between the ER and chloroplasts in leaves, it is feasible that plants possess mechanisms to minimize fatty acid compositional changes in PL to maintain membrane function.

In addition to their presence in leaves and seeds, lipids are also deposited both externally on the pollen surface as a complex mixed polymer termed exine, as well as internally in the form of lipid droplets, which consist mainly of storage TAG (Stanley and Linskens, 1974; Piffanelli *et al.*, 1998; Murphy, 2006). Our GUS fusion results (Fig. 2) demonstrated that within floral tissues, *GPAT9* was expressed preferentially in pollen grains, suggesting a possible role in the production of these pollen lipids. While exine formation did not appear to be affected in GPAT9-OE lines, we found that the number of intracellular lipid droplets was significantly increased, and the droplets themselves seemed to be larger, compared to wild-type plants (Fig. 5B, C). Taken together, these results point to a role for GPAT9 in TAG biosynthesis within pollen grains as has been found previously to be the case for the Kennedy pathway DGAT1 in Arabidopsis (Zhang *et al.*, 2009).

Unexpectedly, we also found an increase in lipid droplet production in our GPAT9-RNAi lines compared to wild type, although this enhancement was significantly less than that observed in the overexpression lines (Fig. 5C). These results were in line with the fact that we saw no abnormalities in pollen morphology or viability within these lines (Fig. 5A), but do not appear to corroborate the precise role of GPAT9 in lipid biosynthesis implied by our overexpression lines. Intriguingly, a similar phenomenon was noted previously following the knockout of *GPAT1* in Arabidopsis (Zheng *et al.*, 2003). GPAT1 was found to play a crucial role in pollen development and morphology, whereby the size of lipid droplets within pollen grains was found to be significantly increased compared to wild type (Zheng *et al.*, 2003). These findings could possibly be attributed to an enhancement or activation of other *GPAT* genes, which in turn over-compensated for down-regulation of *GPAT1* and *GPAT9*. Alternatively, other lipid biosynthetic pathways may be capable of taking over in such instances, as has been found to be the case for the acyl-CoA-dependent Kennedy pathway DGAT1 and acyl-CoA-independent phospholipid:diacylglycerol acyltransferase in pollen lipid biosynthesis, which requires both genes encoding these TAG-biosynthetic enzymes to be down-regulated/inactivated for pollen lipid biosynthesis to be affected (Zhang *et al.*, 2009).

Significant increases and decreases in the content of seed oil, as well as seed size, were also noted in both constitutive and seed-specific *GPAT9* overexpression and RNAi lines, respectively, compared to wild type (Supplementary Figs S4–S8). These findings confirm the recent Arabidopsis *GPAT9* knockdown results obtained by Shockey *et al.* (2016) and demonstrate that this gene also plays a role in seed oil biosynthesis. Taken together, our results provide clear evidence that *GPAT9* contributes to glycerolipid biosynthesis in various tissue types via a role in the Kennedy pathway.

## Supplementary data

Supplementary data are available at *JXB* online.

Data S1. Description of supplementary results.

Table S1. Comparison of transcript levels from selected lipid biosynthetic genes.

Figure S1. Quantitative real-time RT-PCR analysis of Arabidopsis *GPAT9* expression.

Figure S2. Schematic representations of experimental plant transformation constructs.

Figure S3. Confirmation of alterations in *GPAT9* expression in siliques from GPAT9-OE and GPAT9-RNAi homozygous lines compared to wild type.

Figure S4. Seed weight and seed area in homozygous GPAT9-OE, GPAT9-RNAi and wild-type lines.

Figure S5. Oil content and fatty acid composition of T_2_ GPAT9-OE, GPAT9-SS-OE and wild-type seeds.

Figure S6. Seed oil content and composition in homozygous T_3_ GPAT9-OE and wild-type lines.

Figure S7. Oil content and fatty acid composition of T_2_ GPAT9-RNAi, GPAT9-SS-RNAi and wild-type seeds.

Figure S8. Seed oil content and composition in homozygous T_3_ GPAT9-RNAi and wild-type lines.

Figure S9. Growth rates of GPAT9-OE, GPAT9-RNAi and wild-type seedlings.

Figure S10. Confirmation of alterations in *GPAT9* expression in GPAT9-SS-OE and GPAT9-SS-RNAi lines compared to wild type.

Supplementary Data

## Abbreviations:

DAF: days after flowering
DAP: days after planting
DCA: dicarboxylic acid
DGAT: diacylglycerol acyltransferase
ER: endoplasmic reticulum
FA: fatty acid
G3P: *sn*-glycerol-3-phosphate
GPAT: glycerol-3-phosphate acyltransferase
GUS: β-glucuronidase
LPA: lysophosphatic acid
PC: phosphatidylcholine
PL: polar lipids
PP2AA3: *PROTEIN PHOSPHATASE 2A SUBUNIT 3*
TAG: triacylglycerol.
