# Supplementary material for: Arabidopsis GPAT9 contributes to synthesis of intracellular glycerolipids but not surface lipids
Source: J Exp Bot. 2016 Jun 20;67(15):4627–38. doi: 10.1093/jxb/erw242 (PMC4973736; doi:10.1093/jxb/erw242)
Supplement: Supplementary Data [file supp_67_15_4627__index.html]

Arabidopsis GPAT9 contributes to synthesis of intracellular glycerolipids but not surface lipids — Arabidopsis GPAT9 contributes to synthesis of intracellular glycerolipids but not surface lipids — Supplementary Data 

# Arabidopsis GPAT9 contributes to synthesis of intracellular glycerolipids but not surface lipids

## Supplementary Data

Data files

- Supplementary\_figures\_S1\_S10.pdf - Supplementary Data
- supplementary\_table\_S1.xlsx - Supplementary Data
