# Supplementary material for: Arabidopsis GPAT9 contributes to synthesis of intracellular glycerolipids but not surface lipids
Source: J Exp Bot. 2016 Jun 20;67(15):4627–38. doi: 10.1093/jxb/erw242 (PMC4973736; doi:10.1093/jxb/erw242)
Supplement: Supplementary Data [file supp_erw242_Supplementary_figures_S1_S10.pdf]

Supplementary Data S1

Over-expression of *GPAT9* in Arabidopsis increases seed oil content and alters the fatty acid composition of seed oil

Constitutive over-expression of the *GPAT9* coding sequence in Arabidopsis resulted in a small but significant 2.8% relative increase in oil content on a per weight basis in T_2 transgenic seeds compared to wild type (Fig. S4A). To ascertain that any changes in lipid production in transgenic seeds were not a result of phenotypic alterations in vegetative or floral tissues, we also produced lines that over-expressed *GPAT9* in a seed-specific manner (GPAT9-SS-OE; Fig. S1). As was the case for the constitutive GPAT9-OE lines (Fig. S2), over-expression of *GPAT9* was confirmed in developing siliques (14 DAF) from a selection of T_1 GPAT9-SS-OE lines via quantitative real-time RT-PCR, whereby *GPAT9* transcripts were detected at levels enhanced by 682.4% to 3137.7% compared to wild-type levels (Fig. S8A). As was the case for T_2 GPAT9-OE seeds, T_2 GPAT9-SS-OE seeds also exhibited a small but significant 3.0% relative increase in oil content compared to wild-type seeds (Fig. S4B).

To further verify these changes in seed oil content in *GPAT9* over-expression lines, T_3 seeds from two homozygous GPAT9-OE lines with confirmed over-expression of *GPAT9* were similarly analyzed. Interestingly, while oil content analyses of these lines demonstrated small relative increases in seed oil content as a percentage of weight (1.6% and 2.9%, respectively), these changes were not significant (Fig. S5A); a result which could possibly be attributed to the relatively small sample size of T_3 homozygous plants compared to T_2 lines and the inherent variability of seed oil content in Arabidopsis, which can make detecting statistically valid changes in oil content difficult (Li et al. 2006). However, due to the increase in seed size in GPAT9-OE lines, when seed oil content was measured on a per seed basis rather than on a per weight basis, a significant increase in mean oil content was noted in both transgenic lines, corresponding to 11.7% (GPAT9-OE-6) and 12.2% (GPAT9-OE-7) relative increases compared to wild-type plants (Fig. S5B).

Significant alterations in the FA composition of seed oil from both GPAT9-OE and GPAT9-SS-OE lines were also evident. Overall, the seed oil of transgenic lines exhibited reductions in 20:1, as well as increases in 18:1, and 22:0 (Fig. S4 and Fig. S5C).

Down-regulation of *GPAT9* in Arabidopsis decreases seed oil content and alters the fatty acid composition of seed oil

Constitutive down-regulation of *GPAT9* in Arabidopsis using RNAi resulted in a significant 5.3% relative decrease in oil content on a per weight basis in T_2 transgenic seeds compared to wild type (Fig. S6A). Similarly, seed-specific GPAT9-SS-RNAi lines (Fig. S1), where *GPAT9* transcripts were reduced in T_1 developing siliques (14 DAF) in a selection of lines by between 32.3% and 29.6% compared to wild-type levels (Fig. S9B), exhibited a significant 8.9% relative decrease in oil content compared to wild-type lines (Fig. S6B).

To provide further verification of these reductions in seed oil content in GPAT9 RNAi lines, we also analyzed lipids from T_3 seeds from two independent homozygous GPAT9-RNAi lines with confirmed down-regulation of *GPAT9*. As was the case for both constitutive GPAT9-RNAi and seed-specific GPAT9-SS-RNAi T_2 seeds, oil content analyses of GPAT9-RNAi T_3 seeds demonstrated a significant decrease in seed oil content (relative decreases of 7.3% and 4.2% for GPAT9-RNAi-10 and GPAT9-RNAi-21 lines, respectively, compared to wild type) as a percentage of weight (Fig. S7A). Similarly, when seed oil content was measured on a per seed basis rather than on a per weight basis, even greater decreases in mean oil content were noted in both transgenic lines, corresponding to 20.6% (GPAT9-RNAi-10) and 8.0% (GPAT9-RNAi-21) relative reductions compared to wild-type lines (Fig. S7B).

Significant alterations in FA composition of the seed oil of both GPAT9-RNAi and GPAT9-SS-RNAi lines were also evident. Overall, the seed oil of transgenic lines exhibited reductions in 16:0, 18:2, 20:1, 22:0 and 22:1 FAs, as well as increases in 18:0, 18:1, and 18:3 (Fig. S6 and Fig. S7C).

References

Li Y, Beisson F, Pollard M, Ohlrogge J. 2006. Oil content of Arabidopsis seeds: the influence of seed anatomy, light and plant-to-plant variation. Phytochemistry 67, 904-915.

Supplemental figures

Title: **Arabidopsis GPAT9 contributes to synthesis of intracellular glycerolipids but not surface lipids**

Authors:
Stacy D. Singer [1,*], Guanqun Chen [1, *, †], Elzbieta Mietkiewska[1], Pernell Tomasi [2], Kethmi Jayawardhane [1], John M. Dyer[2], and Randall J. Weselake [1, §]

Figure S1: Quantitative real-time RT-PCR analysis of Arabidopsis *GPAT9* expression

Figure S2: Schematic representations of experimental plant transformation constructs.

Figure S3: Confirmation of alterations in *GPAT9* expression in siliques from GPAT9-OE and GPAT9-RNAi homozygous lines compared to wild type.

Figure S4: Seed weight and seed area in homozygous GPAT9-OE, GPAT9-RNAi and wild-type lines.

Figure S5: Oil content and fatty acid composition of T_2 GPAT9-OE, GPAT9-SS-OE and wild-type seeds.

Figure S6: Seed oil content and composition in homozygous T_3 GPAT9-OE and wild-type lines.

Figure S7: Oil content and fatty acid composition of T_2 GPAT9-RNAi, GPAT9-SS-RNAi and wild-type seeds.

Figure S8: Seed oil content and composition in homozygous T_3 GPAT9-RNAi and wild-type lines.

Figure S9: Growth rates of GPAT9-OE, GPAT9-RNAi and wild-type seedlings.

Figure S10: Confirmation of alterations in *GPAT9* expression in GPAT9-SS-OE and GPAT9-SS-RNAi lines compared to wild type.

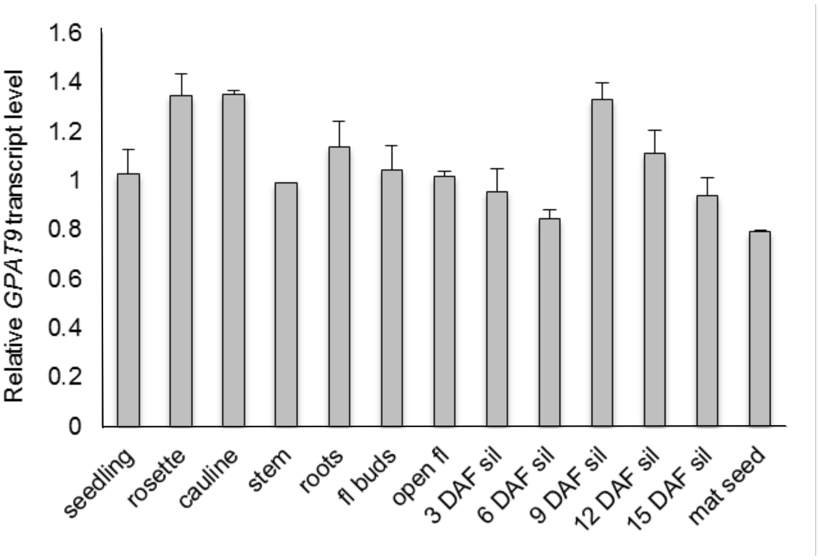

Fig. S1. Arabidopsis *GPAT9* expression analyses with quantitative real-time RT-PCR analysis of Arabidopsis Col-0 tissues at numerous developmental stages. Three technical replicates were carried out in each case. Blocks represent the mean *GPAT9* transcript level of two biological replicates relative to levels of the internal control transcript, *PP2AA3*. Bars denote standard errors. DAF, days after flowering; fl, flower; mat, mature; sen, senescing; sil, siliques.

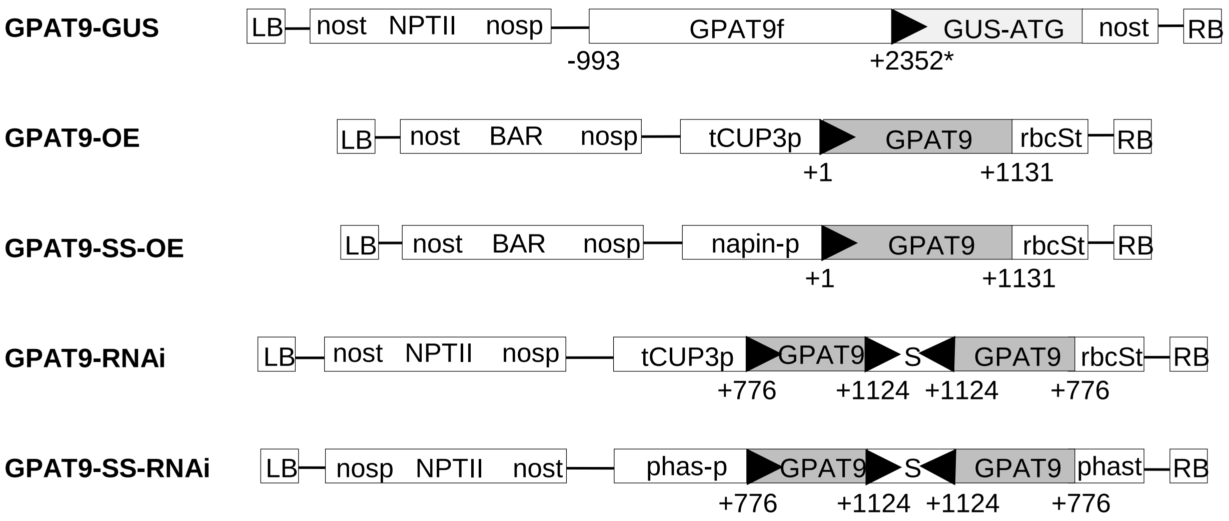

Fig. S2. Schematic representations (not to scale) of experimental plant transformation constructs utilized in this study. Numbers indicate nucleotides relative to the Arabidopsis *GPAT9* start codon (+1). Numbers within the GPAT9-GUS vector representation denote nucleotides of genomic sequence, while those of the remaining vectors indicate coding sequence. Arrows indicate the direction of transcription in each case. BAR, phosphinothricin acetyltransferase; GPAT9, Arabidopsis *GPAT9* sequence; LB, left T-DNA border; napin-p, *Brassica napus Napin* promoter; nosp, *Nopaline synthase* promoter; nost, *Nopaline synthase* transcriptional terminator; NPTII, *Neomycin phosphotransferase II*; phas-p, *Phaseolus vulgaris Phaseolin* promoter; phast, *Phaseolin* transcriptional terminator; RB, right T-DNA border; rbcSt, *Pisum sativum Ribulose-1,5-bisphosphate carboxylase* transcriptional terminator; S, intronic spacer; tCUP3p, tobacco *tCUP3* constitutive promoter.

A

B

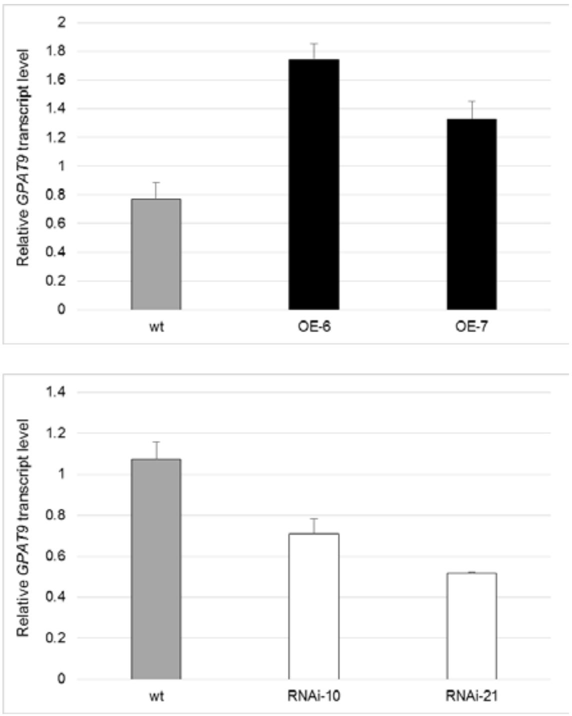

Fig. S3. Confirmation of alterations in *GPAT9* expression in siliques from GPAT9-OE and GPAT9-RNAi homozygous lines compared to wild type. Quantitative real-time RT-PCR analysis of *GPAT9* expression in homozygous T_2 siliques (14 DAF) from GPAT9-OE **(A)**, GPAT9-RNAi **(B)** and wild-type lines. Three technical replicates were carried out in each case. Blocks denote mean *GPAT9* transcript levels from three biological replicates relative to the internal control, *PP2AA3*, and bars indicate standard errors.

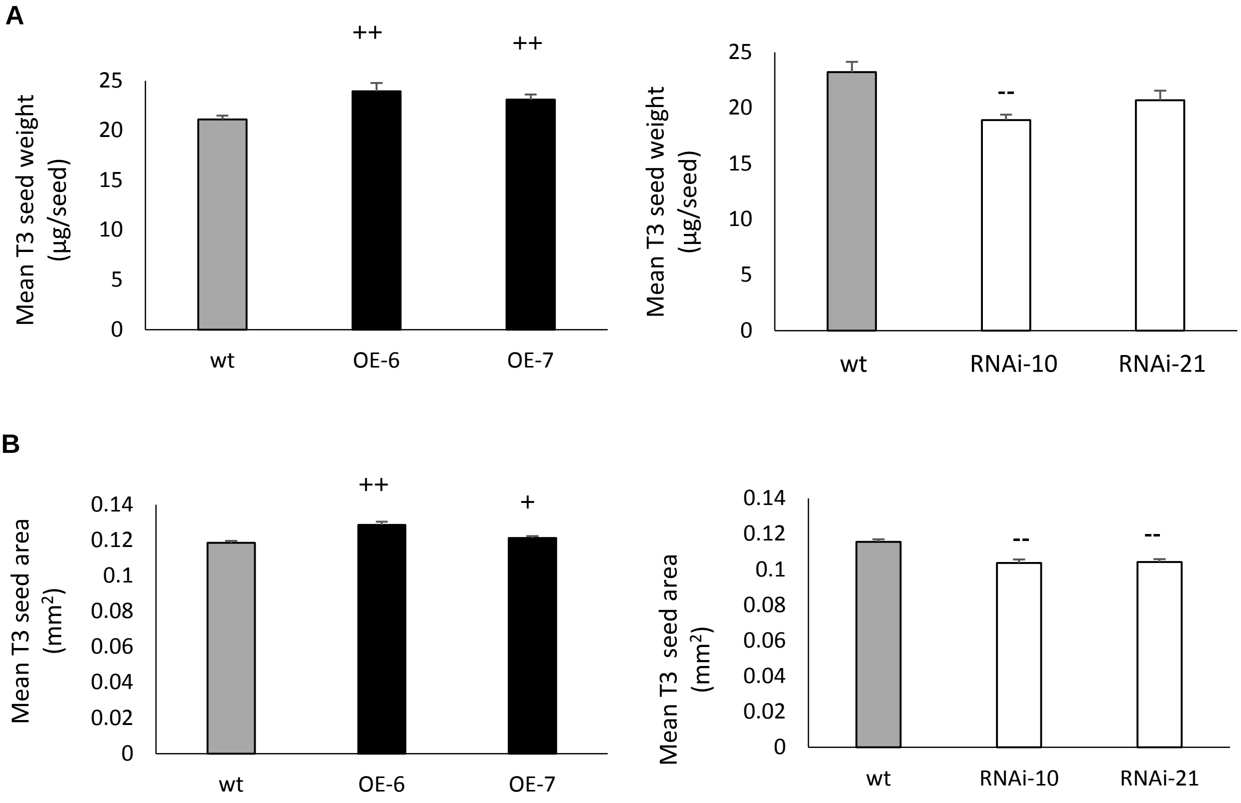

Fig. S4. Seed weight and seed area in homozygous GPAT9-OE, GPAT9-RNAi and wild-type lines. A, Seed weights of T_3 homozygous lines. Blocks indicate mean weights of seeds from wild-type (*n*=14), OE-6 (*n*=5), OE-7 (*n*=7); wild-type (*n*=15), RNAi-10 (*n*=5), and RNAi-21 (*n*=7) lines, with three technical replicates measured in each case. B, Seed areas of T_3 homozygous lines. Blocks indicate mean areas of wild-type (*n*=113), OE-6 (*n*=65), OE-7 (*n*=107); wild-type (*n*=127), RNAi-10 (*n*=59), and RNAi-21 (*n*=81) seeds. In all instances, bars denote standard errors. Significant increases and decreases compared to wild type (as measured by Student's t-test) are indicated by +/++ ($P \leq 0.05/P \leq 0.01$) and - - ($P \leq 0.01$). OE, GPAT9-OE lines, RNAi, GPAT9-RNAi lines, wt, wild type.

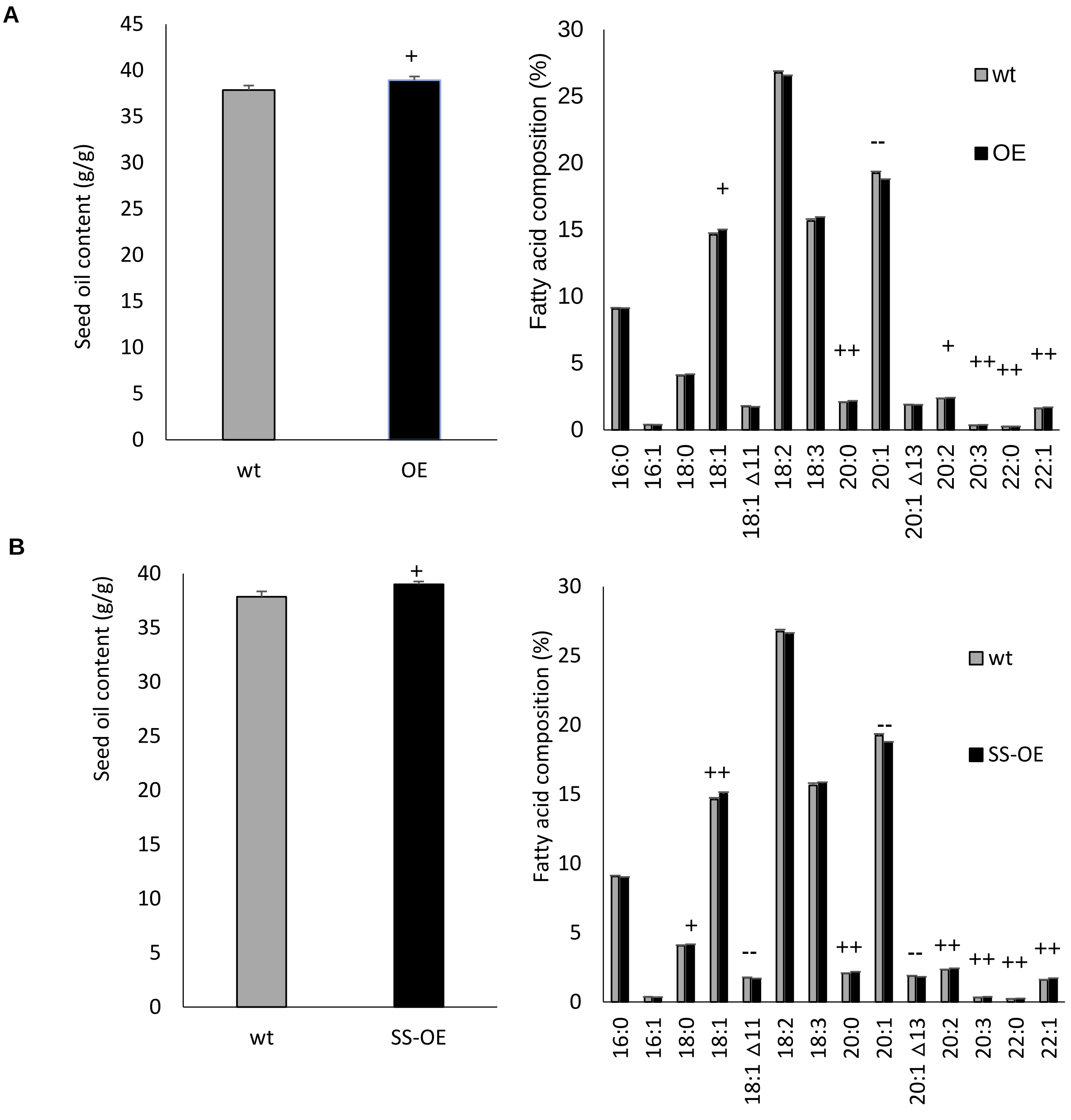

Fig. S5. Oil content and fatty acid composition of T_2 GPAT9-OE, GPAT9-SS-OE and wild-type seeds. A, Mean seed oil content and fatty acid composition in GPAT9-OE lines. Blocks represent mean values of wild-type (*n*=14) and GPAT9-OE (*n*=15) independent lines. B, Mean seed oil content and fatty acid composition in GPAT9-SS-OE lines. Blocks indicate mean values of wild-type (*n*=14) and GPAT9-SS-OE (*n*=15) independent lines. Two technical replicates were carried out for every line analyzed. Bars denote standard errors. Significant increases and decreases compared to wild type (as measured by Student's t-test) are indicated by +/++ ($P \leq 0.05$/$P \leq 0.01$) and - - ($P \leq 0.01$). OE, GPAT9-OE; SS-OE, GPAT9-SS-OE; wt, wild type; TFA, total fatty acid.

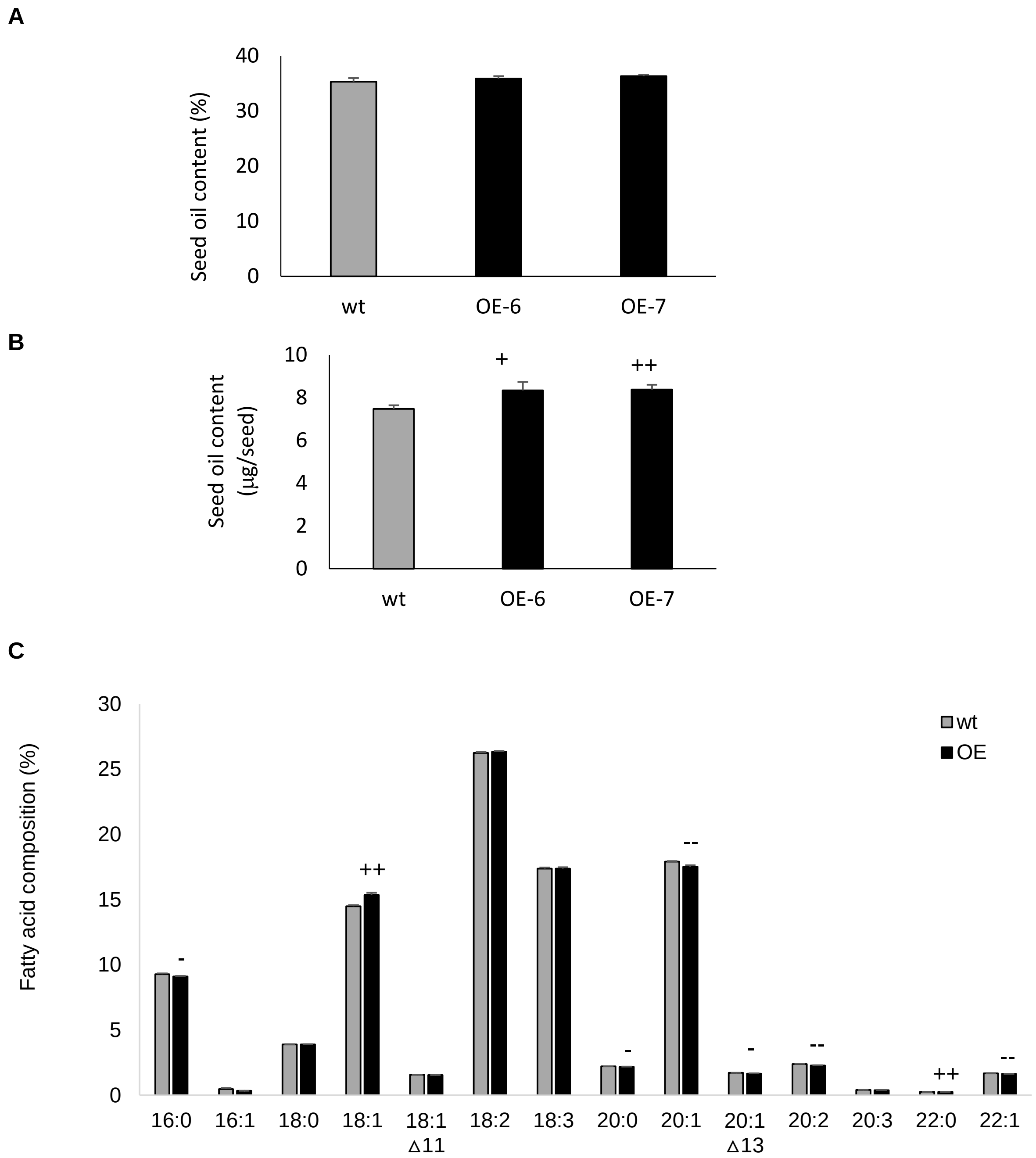

Fig. S6. Seed oil content and composition in homozygous T_3 GPAT9-OE and wild-type lines. A, Seed oil content on a per weight basis. B, Seed oil content on a per seed basis. In the case of oil content analyses, blocks represent mean values from wild-type (*n*=14), GPAT9-OE-6 (*n*=5) and GPAT9-OE-7 (*n*=7) lines. C, Fatty acid composition of seed oil. In the case of fatty acid composition, blocks represent the mean values of wild-type and pooled GPAT9-OE-6 and GPAT9-OE-7 lines. Bars denote standard errors. Significant increases and decreases compared to wild type (as measured by Student's t-test) are indicated by +/++ ($P \leq 0.05$/$P \leq 0.01$) and -/-- ($P \leq 0.05$/$P \leq 0.01$). OE, GPAT9-OE; wt, wild type.

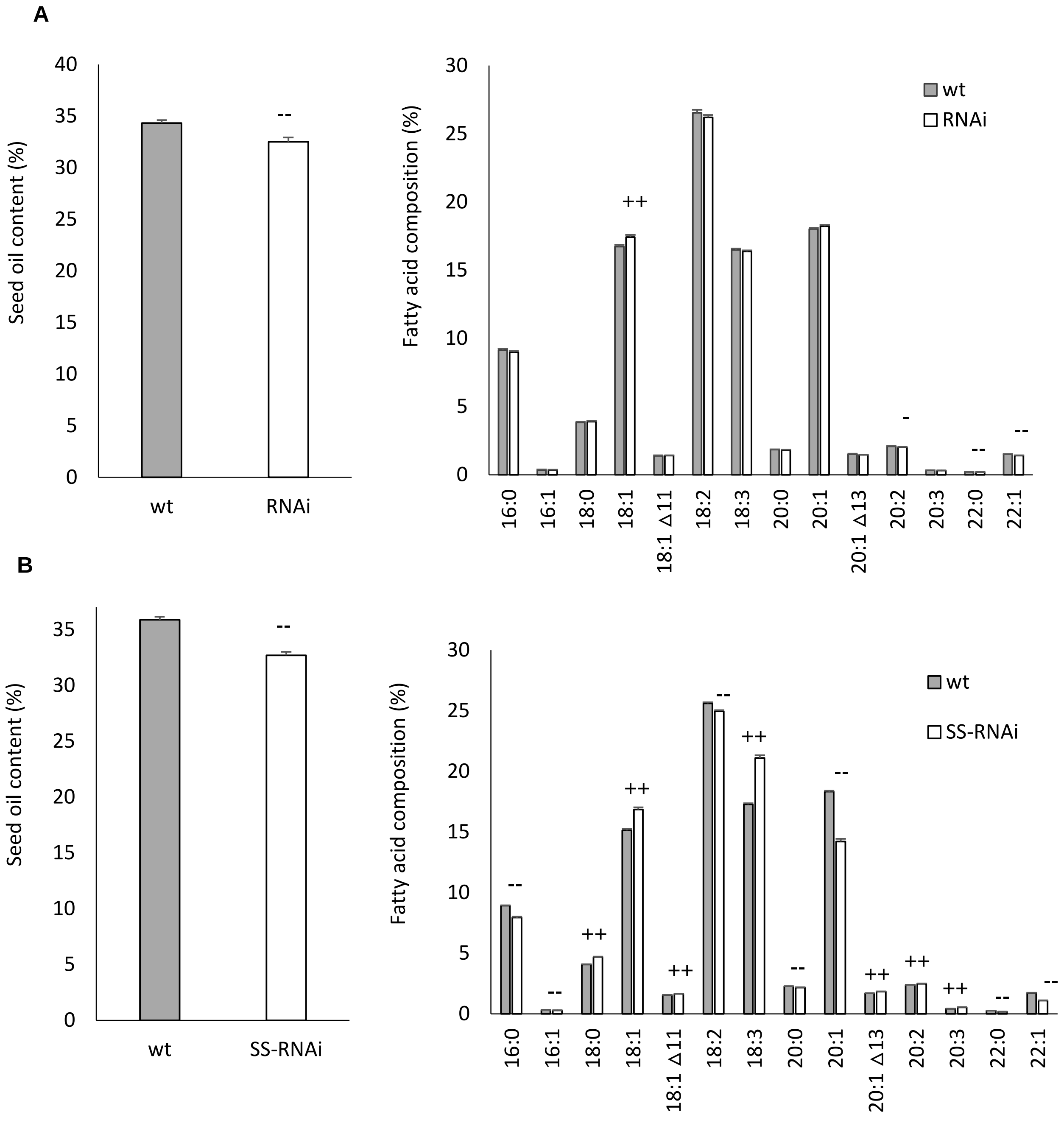

Fig. S7. Oil content and fatty acid composition of T_2 GPAT9-RNAi, GPAT9-SS-RNAi and wild-type seeds. A, Mean seed oil content and fatty acid composition in GPAT9-RNAi lines. Blocks represent mean values of wild-type (*n*=16) and GPAT9-RNAi (*n*=29) independent lines. B, Mean seed oil content and fatty acid composition in GPAT9-SS-RNAi lines. Blocks indicate mean values of wild-type (*n*=25) and GPAT9-SS-RNAi (*n*=25) independent lines. Two technical replicates were carried out for every line analyzed. Bars denote standard errors. Significant increases and decreases compared to wild type (as measured by Student's t-test) are indicated by ++ ($P \leq 0.01$) and -/- - ($P \leq 0.05$/$P \leq 0.01$). RNAi, GPAT9-RNAi; SS-RNAi, GPAT9-SS-RNAi; wt, wild type; TFA, total fatty acids.

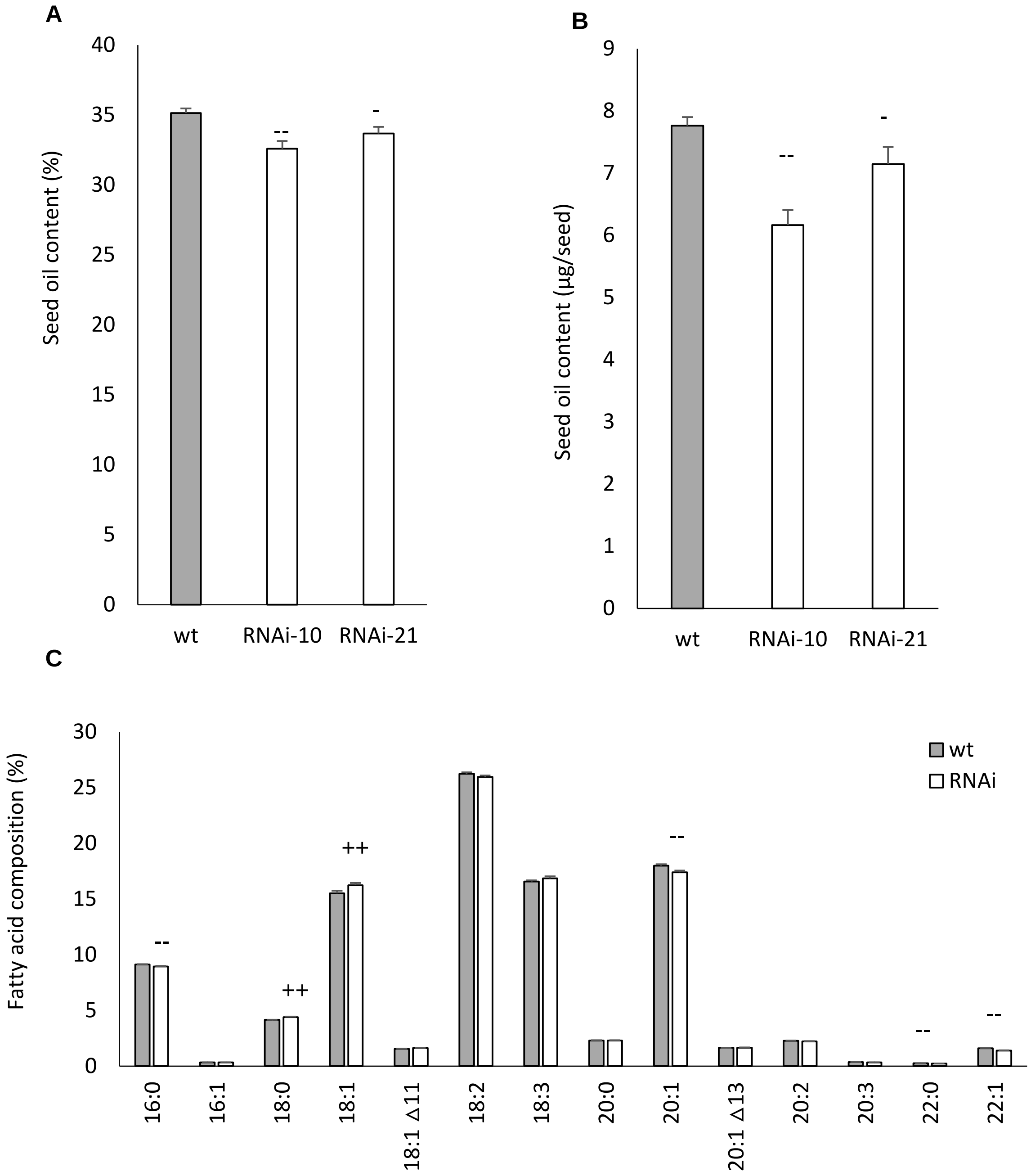

Fig. S8. Seed oil content and composition in homozygous T_3 GPAT9-RNAi and wild-type lines. A, Seed oil content on a per weight basis. B, Seed oil content on a per seed basis.
In the case of oil content, blocks represent mean values from wild-type (*n*=15), GPAT9-RNAi-10 (*n*=5) and GPAT9-RNAi-21 (*n*=7) lines. C, Fatty acid composition of seed oil. In the case of fatty acid composition, blocks represent the mean values of wild-type and pooled GPAT9-RNAi-10 and GPAT9-RNAi-21 lines. Bars denote standard errors. Significant increases and decreases compared to wild type (as measured by Student's t-test) are indicated by ++ ($P \leq 0.01$) and -/- - ($P \leq 0.05$/$P \leq 0.01$). RNAi, GPAT9-RNAi; wt, wild type; TFA, total fatty acids.

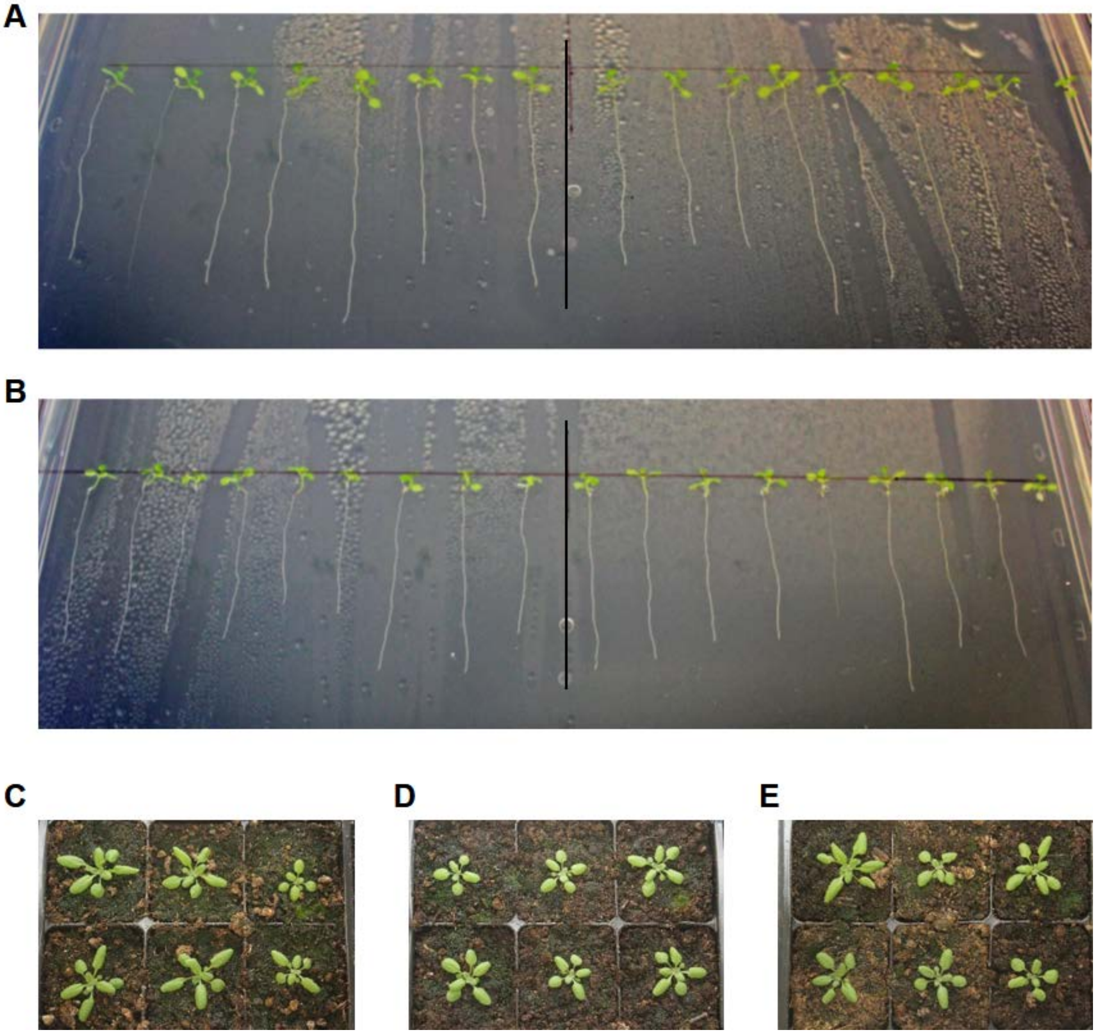

Fig. S9. Growth rates of GPAT9-OE, GPAT9-RNAi and wild-type seedlings.
(A and B) Seedlings grown vertically on solid medium were photographed 15 days post-germination and are representative of two independent experiments. GPAT9-OE **(A)** and GPAT9-RNAi **(B)** seedlings are shown to the left of the black line, while wild-type seedlings are present to the right in both instances.
(C-E) Soil grown wild-type **(C)**, GPAT9-OE **(D)** and GPAT9-RNAi **(E)** lines 17 days post-germination.

A

B

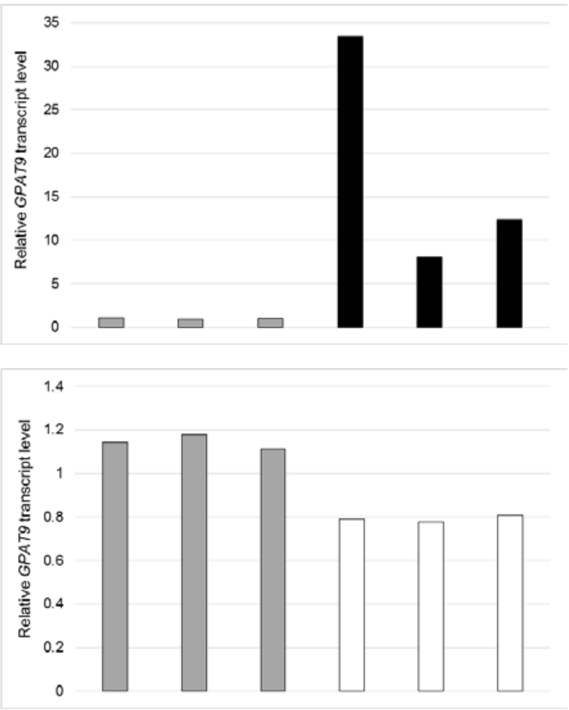

Fig. S10. Confirmation of alterations in *GPAT9* expression in GPAT9-SS-OE and GPAT9-SS-RNAi lines compared to wild type. Quantitative real-time RT-PCR analysis of *GPAT9* expression in T_1 siliques (14 DAF) from independent GPAT9-SS-OE **(A)**, GPAT9-SS-RNAi **(B)** and wild-type lines. Blocks denote mean *GPAT9* transcript levels from three technical replicates relative to the internal control, *PP2AA3*. Gray blocks represent wild-type plants, black blocks represent independent GPAT9-SS-OE lines, and white blocks represent independent GPAT9-SS-RNAi lines.
